# Health-Related Behavior, Profile of Health Locus of Control and Acceptance of Illness in Patients Suffering from Chronic Somatic Diseases

**DOI:** 10.1371/journal.pone.0063920

**Published:** 2013-05-10

**Authors:** Konrad Janowski, Donata Kurpas, Joanna Kusz, Bozena Mroczek, Tomasz Jedynak

**Affiliations:** 1 Department of Psychology, University of Finance and Management, Warsaw, Poland; 2 Department of Family Medicine, Medical University, Wrocław, Poland; 3 Public Higher Medical Professional School, Opole, Poland; 4 Department of Health Sciences, Nursing, Medical University, Wrocław, Poland; 5 Public Health Division, Department of Health Sciences, Pomeranian Medical University, Szczecin, Poland; 6 Department of Clinical Psychology, Faculty of Social Sciences, John Paul II Catholic University of Lublin, Lublin, Poland; The University of Hong Kong, Hong Kong

## Abstract

**Purpose:**

The purpose of the study was to determine health-related behaviors, profile of health locus of control (HLC), and to assess the relationships between these constructs among patients suffering from chronic somatic diseases.

**Material and Methods:**

Three-hundred adult patients suffering from various chronic diseases participated in the study. The patients' mean age was 54.6 years (SD = 17.57).

**Results:**

No statistically significant differences were found between the different clinical groups in health-related behavior, acceptance of illness, internal HLC or chance HLC. Patients with neurologic conditions showed slightly lower powerful others HLC than did some other clinical groups. Health-related behavior was significantly positively related to all three categories of HLC, with most prominent associations observed with powerful others HLC. Only one type of health-related behavior – preventive behavior – correlated significantly and negatively with acceptance of illness. Differences in the frequency of health-related behavior were also found due to gender (women showing more healthy nutritional habits than men), age (older subjects showing more frequent health-promoting behavior), education (higher education was associated with less frequent health-promoting behavior) and marital status (widowed subjects reporting more frequent health-promoting behavior).

**Conclusions:**

Health-related behavior in patients with chronic diseases seems to be unrelated to a specific diagnosis; however it shows associations with both internal and external HLC. Sociodemographic factors are also crucial factors determining frequency of health-related behavior in such patients.

## Introduction

Health-related behavior involves a variety of behavior patterns, actions and habits which bear relevance to health maintenance, restoration or improvement [1]. Since long efforts were made by health care professionals and those responsible for health policies to influence individuals' behavior in such a way so as to minimize risky health-related behavior and maximize preventive or protective health-related behavior [2], [3]. The effects of health-related behavior for actual health are crucial in terms of preventing morbidity and mortality [4]–[6]. However, the significance of health-related behavior is also emphasized with regard to those who have already developed a disease, as patients' behavioral patterns, habits or actions are frequently able to slow down the progress of a disease or to prevent aggravations and relapses [7]–[9]. Various factors have been reported as associated with the likelihood of both preventive and risky health-related behavior, including socioeconomic status, personality, emotional and cognitive factors [10].

Among cognitive factors, generalized beliefs related to health controllability and manageability, usually conceptualized as health locus of control (HLC), were identified as a crucial determinant of health-related behavior [11]. The concept of HLC was developed by Wallston et al. [12], [13] who applied the previously proposed Rotter's construct of *locus of control* [14] to the domain of health. In analogy with Rotter's unidimensional understanding of locus of control, HLC was initially conceptualized as one continuum ranging from the internal to external poles [15]. Later, Wallston et al. [13] proposed that HLC should be viewed as a multidimensional construct, with relatively independent dimensions. These dimensions reflected differences in attributions people can hold about the responsibility for and control of their health. These could be limited to three major categories: (1) internal HLC – the responsibility for one's health is attributed to oneself and to the action one takes with consequences for health; (2) (external) powerful others HLC – the responsibility for one's health is assigned to other people, predominantly medical professionals, who are perceived as those in control of one's health condition; and (3) (external) chance HLC – the responsibility for one's health is believed to depend on uncontrollable factors, such as good/bad luck, or fate [13]. These beliefs are relatively stable characteristics formed in the process of social adaptation and personal experience. They are expressed in the individual's attitudes and subjective norms.

Internal HLC has often been reported to show links with increased self-reliance and independence in taking various health-related behaviors, health-related decisions and health outcomes [16], [17]. In one study among a large sample of young adults from 18 European countries, the odds of five healthy behaviors were 40% greater in individuals with high as compared to those with low internal HLC [18]. In contrast, external HLC was usually reported to be associated with adverse health-related behaviors, such as smoking or excessive alcohol consumption [19] or poorer health outcomes [20]. Chance HLC was also reported as related to unfavorable health-related behaviors, such lower sports activity, fewer medical teeth check-ups, and less health-related information-seeking [21]. Similarly, in a study by Steptoe and Wardle [18], high chance HLC was reported to be associated with more than 20% reductions in the likelihood of 6 healthy behaviors.

However, the results of previous studies have not always been conclusive. Some studies reported that HLC failed to explain variance in health-related habits beyond that explained by basic personality factors, although it was a significant predictor of health attitudes [22]. Internal HLC was reported to be associated also with adverse behaviors, such as more frequent smoking, and unrelated to a range of positive health-related behaviors (sports activity, healthy diet, teeth check-ups, medical check-ups or seeking information about health issues) [21]. In a study of patients with chronic low back pain, none of the scores for the three domains of HLC revealed any significant associations with adherence to therapy [23]. In patients with cancer undergoing mindfulness-based intervention, chance HLC but also internal HLC were found to be significantly lower after treatment [24].

Acceptance of illness is a psychological indicator of the quality of adaptation to life with a disease. Since chronic diseases usually impose a range of limitations on normal everyday functioning and are able to affect quality of life, patients may show difficulties adapting to such limitations and accepting their inevitability [25]. Therefore, patients may reveal different levels of acceptance of illness, which reflects how well they tolerate the burden of the disease [26], [27]. Acceptance of illness may affect the likelihood of health-related behavior, through modification of motivation to undertake particular actions [28]. For instance, patients with high acceptance of illness may feel motivated to undertake or continue behavior which helps them maintain the lowest possible burden of the disease. On the other hand, high acceptance of the disease may be related to satisfaction with the *status quo* and no need for further effort may be perceived as required to improve one's situation.

The purpose of this study was to evaluate desirable health-related behaviors revealed by patients suffering from chronic diseases. In particular, the study aimed at verifying whether patients with different categories of diseases show similar levels of positive health-related behaviors and whether other psychological factors, such as health locus of control and acceptance of illness, can affect the reported frequencies of health-related behaviors. Additionally, the effects of sociodemographic variables on health-related behaviors were controlled.

## Materials and Methods

### Participants

The sample consisted of 300 adult patients suffering from various chronic diseases. Mean age of the patients was 54.6 (SD = 17.57) years, ranging from 18 to 85. The sociodemographic data for the sample are presented in Table 1.

**Table 1:** Sociodemographic data of the investigated patients with chronic diseases.

	*N*	*%*
Gender		
Women	173	57.7
Men	127	42.3
Place of residence		
Rural	101	33.7
Small towns	155	51.6
Major cities	44	14.7
Education		
Primary	49	16.3
Vocational	97	32.3
Secondary	81	27
Post-secondary	17	5.7
Higher	56	18.7
Marital status		
Single	63	21
Married	169	56.3
Divorced	15	5
Widowed	53	17.7

### The Study Procedure

Patients were recruited from two internal and neurological clinics in two minor towns in Poland. The patients were included into the sample if they had a physician-made single diagnosis of a chronic disease falling into six broad clinical categories: (1) respiratory diseases, (2) urinary diseases, (3) circulatory system diseases, (4) locomotor diseases, (5) diabetes, or (6) chronic neurological conditions. Inclusion criteria were: age ≥18 years, a single chronic disease lasting for at least 12 months prior to the study, and native knowledge of Polish necessary to complete the questionnaires. Patients with the following criteria were excluded: active cancer disease (defined as cancer diagnosis under current treatment with radiotherapy or chemotherapy), moderate to severe dementia, severe mental disorders, other problems that disable active participation in the study (e.g. non-Polish speaking patients, severe vision problems). Consecutive patients from each clinical category were invited to take part in the study. Recruitment to a particular clinical group was complete when the limit of 50 patients was reached in the group. The participation was anonymous and written informed consent was obtained from all patients. The patients were given a battery of questionnaires to complete at home and returned them in a pre-addressed and stamped envelope. If the consent form was not signed and returned, extra patients were recruited again.

The study protocol was approved by the Bioethics Committee of Wrocław Medical University and registered as KB-19/2012.

### Methods

Three questionnaires were used in the study: Health-Related Behaviors Inventory (HRBI), The Multidimensional Health Locus of Control Scale (MHLCS) and Acceptance of Illness Scale (AIS).

Health-Related Behaviors Inventory allows evaluating the frequency of four categories of health-related behaviors: healthy eating habits, preventive behaviors, positive mental attitude, and healthy practices. Healthy eating habits include behaviors related to the choice of healthy foods in everyday diet. Preventive behaviors involve compliance with health-related guidelines and with the possessed knowledge on health and disease. Positive mental attitude relates to behaviors such as avoiding excessively strong emotions, tensions, and stressful or upsetting situations. Healthy practices include desirable sleep, entertainment and physical activity habits [29]. The total score of HRBI is obtained by summing up the scores for each subscale. Higher scores indicate higher frequency of a given category of health-related behaviors. The authors of this instrument report satisfactory Cronbach's alpha reliability coefficients for the subscales, ranging from 0.60 to 0.64. High reliability is reported for the total score:.85 and.88 for internal consistency (Cronbach's alpha) and stability (test-retest), respectively. Validity of this instrument was tested in terms of theoretical (factor), convergent and divergent validity, with the results demonstrating that the measurement is valid [29].

The Multidimensional Health Locus of Control Scale was used to assess the profile of HLC. This questionnaire is a brief self-report measure providing scores for internal and external HLC: The category of external HLC is subdivided into powerful others HLC, and chance HLC [30]. Higher scores are indicative of a more intense HLC in a given category. In this study, the Polish version of the instrument was used. Cronbach's alpha reliabilities for the Polish version are satisfactory and range from.54 to.74, depending on the subscale and the investigated sample. Validation data were also provided for the Polish version of the instrument [29].

The Acceptance of Illness Scale is an eight item self-report measure designed to evaluate adjustment to a chronic illness [31]. The items are worded in such a way that they describe negative consequences of illness, such as limitations, dependence on others, or lowered self-esteem. The total score is calculated as a sum of scores for each item. Higher scores indicate higher acceptance and better adjustment to illness. Reliability of the scale was reported as satisfactory to high, with Cronbach's alpha  = .85 and test-retest reliability.64 [29].

### Statistical Methods

One-way analysis of variance was performed to test the differences on the analyzed variables between the clinical groups. Statistically significant findings obtained in the ANOVA analysis were compared by means of *post hoc* tests with Bonferroni corrections for multiple comparisons. In order to evaluate the strength of the relationships between the variables, two-sided Pearson's *r* correlation coefficients were calculated. In order to determine the relationship between gender and the scores on the questionnaires, Student's *t*-tests were performed and Bonferroni correction for multiple comparisons was applied. Statistical significance was established at the level of *P*≤.05. The data were processed using SPSS 19.0 software.

## Results

### Between-Group Differences in Health-Related Behaviors, HLC, and Acceptance of Illness

The global index of health-related behaviors was found to be highest in the sample of patients with diabetes (*M* = 90.18, *SD* = 16.02) and lowest in patients with circulatory diseases (*M* = 81.12, *SD* = 17.68). Although the ANOVA *F* test showed a statistically significant inter-group difference, *post-hoc* tests corrected for multiple comparisons did not yield statistically significant differences. Similarly, the samples of patients with different diagnostic categories did not differ significantly with respect to any of the measured subtype of health-related behaviors (Table 2).

**Table 2:** Descriptive statistics for health-related behaviors, health locus of control and acceptance of illness in the samples with different diagnoses.

	Health-related behaviors					Health locus of control			
Sample	Total score	Healthy eating habits	Preventive behaviors	Positive mental attitude	Healthy practices	Internal	Powerful others	Chance	Acceptance of illness
	*M* (*SD*)	*M* (*SD*)	*M* (*SD*)	*M* (*SD*)	*M* (*SD*)	*M* (*SD*)	*M* (*SD*)	*M* (*SD*)	*M* (*SD*)
1	81.84 (16.87)	19.06 (5.12)	20.56 (5.36)	22.28 (4.65)	19.94 (5.16)	25.68 (5.45)	25.98 (6.65)	24.32 (7.08)	29.09 (8.46)
2	81.12 (17.68)	18.84 (5.61)	20.56 (5.30)	21.40 (5.08)	20.32 (5.70)	28.30 (5.18)	27.28 (6.46)	26.72 (5.54)	27.78 (9.86)
3	85.14 (15.97)	19.56 (4.93)	21.50 (5.27)	22.82 (5.00)	21.26 (4.84)	26.21 (5.23)	26.63 (5.86)	24.40 (6.64)	26.63 (8.55)
4	87.12 (19.18)	20.44 (5.76)	22.84 (5.35)	22.44 (5.43)	21. 40 (5.59)	25.98 (6.46)	28.00 (6.14)	27.26 (5.99)	28.62 (9.26)
5	90.18 (16.02)	21.26 (4.90)	22.74 (4.96)	23.44 (4.65)	22.74 (4.67)	27.92 (6.00)	28.08 (6.45)	25.76 (7.09)	25.76 (10.34)
6	81.38 (14.30)	18.34 (5.34)	20.88 (4.97)	21.82 (3.98)	20.34 (4.61)	25.00 (5.53)	24.12 (7.44)	24.72 (5.75)	27.02 (8.92)
One-way ANOVA	*F*(5,294) = 2.42	*F*(5,294) = 2,12	*F*(5,294) = 2,02	*F*(5,294) = 1,12	*F*(5,294) = 2.02	*F*(5,292) = 2.66	*F*(5,292) = 2.61	*F*(5,292) = 1.92	*F*(5,288) = 0.89
	*P* = 0.036	*P* = 0.063	*P* = 0.075	*P* = 0.349	*P* = 0.076	*P* = 0.023	*P* = 0.025	*P* = 0.090	*P* = 0.488
*Post hoc* tests with Bonferroni correction for multiple comparisons	n. s.	n. s.	n. s.	n. s.	n. s.	n. s.	Differences between samples: 6–4 (*P* = 0.039) 6–5 (*P* = 0.048)	n. s.	n. s.

Samples: 1- patients with respiratory diseases; 2- patients with circulatory diseases; 3- patients with locomotor diseases; 4- patients with urinary system diseases; 5- patients with diabetes; 6- patients with nervous system diseases.

M – mean, SD – standard deviation, HLC – health locus of control, n. s. – non significant.

The samples of patients with different diagnostic categories differed statistically significantly with respect to powerful others HLC, with the group of neurological patients scoring significantly lower than patients with diabetes and patients with diseases of the urinary system. No statistically significant differences between the compared groups were found with respect to internal and chance HLC or acceptance of illness (Table 2).

### Correlations between Health-Related Behavior, Profile of Health Locus of Control and Acceptance of Illness

The analysis of the correlations computed for the total sample demonstrated positive associations between health-related behavior categories and types of HLC. The strongest Pearson's *r* coefficients occurred between external (powerful others) HLC and preventive behaviors, positive mental attitude, health practices and healthy eating habits. All these coefficients were above.30. In the case of internal HLC, statistically significant correlations were observed with positive mental attitude, preventive behaviors and healthy practices, although these associations were relatively weaker. There was no statistically significant correlation between internal HLC and healthy eating habits. Weak positive correlations were also found between chance HLC and all subtypes of health-related behaviors. Only one statistically significant correlation was found for acceptance of illness and it was a negative correlation with preventive behaviors (Table 3). No statistically significant associations were found between types of HLC and acceptance of illness.

**Table 3:** Pearson's *r* correlation coefficients between health-related behaviors, health locus of control and acceptance of illness.

Variables	Internal HLC	Powerful others HLC	Chance HLC	Acceptance of illness
Total sample				
Health-related behaviors - total score	.20***	.49***	.23***	−.10
Healthy eating habits	.11	.34***	.17**	−.08
Preventive behaviors	.17**	.48***	.18**	−.12*
Positive mental attitude	.22***	.45***	.21***	−.05
Healthy practices	.14*	.36***	.20**	−.07
Patients with respiratory diseases				
Health-related behaviors - total score	.12	.54***	.12	−.15
Healthy eating habits	.06	.34*	.01	−.05
Preventive behaviors	.14	.55***	.19	−.21
Positive mental attitude	.21	.46***	.15	−.19
Healthy practices	.00	.44***	.05	−.07
Patients with circulatory diseases				
Health-related behaviors - total score	.31*	.56***	.39**	.02
Healthy eating habits	.33*	.43**	.42**	−.01
Preventive behaviors	.28*	.52***	.25	−.01
Positive mental attitude	.28*	.45***	.36*	.00
Healthy practices	.14	.43**	.26	.10
Patients with locomotor diseases				
Health-related behaviors - total score	.28	.41**	−.03	−.26
Healthy eating habits	.08	.21	−.14	−.29*
Preventive behaviors	.33*	.29*	−.13	−.17
Positive mental attitude	.31*	.48***	.16	−.24
Healthy practices	.13	.31*	.03	−.15
Patients with the urinary system diseases				
Health-related behaviors - total score	.27	.55***	.33*	−.17
Healthy eating habits	.18	.42**	.31*	−.16
Preventive behaviors	.21	.51***	.24	−.29*
Positive mental attitude	.22	.60***	.24	.02
Healthy practices	.33*	.39**	.35*	−.13
Patients with diabetes				
Health-related behaviors - total score	.24	.53***	.45***	.18
Healthy eating habits	.11	.39**	.34*	.29*
Preventive behaviors	.17	.52***	.40**	.12
Positive mental attitude	.21	.44***	.37**	.10
Healthy practices	.32*	.44***	.38**	.08
Patients with neurological diseases				
Health-related behaviors - total score	−.14	.31*	.05	−.22
Healthy eating habits	−.19	.16	.00	−.27
Preventive behaviors	−.07	.43**	.05	−.15
Positive mental attitude	.14	.26	−.05	.05
Healthy practices	−.26	.08	.12	−.24

* *P*≤.05 ** *P*≤.01 ***P≤.001.

The same analysis carried out separately for the subsamples of patients with different categories of diseases yielded a slightly more complex picture of the associations. Generally, a similar pattern of positive correlations between health-related behaviors and internal, powerful others and chance HLC was observed in all the subsamples divided by condition type. Additionally, in all the subsamples, powerful others HLC showed the strongest correlations with health-related behavior, whereas relatively weaker associations were observed for internal and chance HLC.

Some interesting inter-group differences also emerged from this analysis. While powerful others HLC was significantly correlated with at least some of health-related behaviors in all the subsamples, internal and chance HLC were found to correlate significantly with health-related behavior only in some of the subsamples. Internal HLC correlated significantly with health-related behavior in patients with diabetes, circulatory, urinary and locomotor diseases, no such correlations were found in patients with respiratory and neurological diseases. Chance HLC correlated significantly with health-related behavior in patients with diabetes, urinary and circulatory diseases, and no such correations were observed in the remaining subsamples.

Acceptance of illness was unrelated to health-related behavior in patients with circulatory, respiratory and neurological diseases. In patients with locomotor and urinary diseases, acceptance of illness was negatively associated with health-related behavior, whereas a positive correlation between these variables was observed in patients with diabetes.

### Health-Related Behaviors and Sociodemographic Variables

The comparison of the mean scores for healthy eating habits obtained for women (*M* = 20.26, *SD* = 5.12) and men (*M* = 18.66, *SD* = 5.49) yielded a statistically significant difference. Women reported healthy eating habits significantly more frequently than men did (*t*(298) = 2.59, *P* = .010). No statistically significant gender differences were found for other types of health-related behaviors.

A statistically significant positive correlation was observed between age and the total score on health-related behaviors (*r*(298) = .41, *P*≤.001). Similarly, statistically significant correlations with age were found for healthy practices (*r*(298) = .39, *P*≤.001), positive mental attitude (*r*(298) = .35, *P*≤.001), preventive behaviors (*r*(298) = .33, *P*≤.001), and healthy eating habits (*r*(298) = .29, *P*≤.001).

Analysis of variance in health related behaviors was performed between subgroups with different educational level. Statistically significant differences were found for the total score of health-related behaviors (*F*(4,295) = 2.84, *P* = .025), with patients with higher education reporting significantly less frequent health-related behaviors than those with primary (*post hoc P* = .047) and vocational (*post hoc P* = .036) education. In particular, patients with higher education revealed statistically significantly less frequent preventive behaviors (*F*(4,295) = 3.45, *P* = .009) than patients with primary and vocational education (*post hoc P* = .034 and.020, respectively). Patients with higher education also revealed statistically significantly (*F*(4,295) = 3.04, *P* = .009) lower scores on positive mental attitude than those with vocational education (*post hoc P* = .018). Similarly, healthy practices were also found significantly less frequent (*F*(4,295) = 3.55, *P* = .008) in patients with higher education than in those with primary and vocational education (*post hoc P* = .030 and.009, respectively).

No statistically significant differences were found on health-related behaviors between subgroups of patients with different place of residence.

Analysis of variance for marital status revealed statistically significant differences for the total score of health-related behaviors (*F*(3,296) = 6.10, *P* = .000), with widowed patients reporting such behaviors significantly more frequently than single, married and divorced patients (*post hoc P* = .001,.043, and.019, respectively). In particular, widowed patients exhibited significantly more frequent (*F*(3,296) = 6.30, *P* = .000) preventive behaviors than single (*post hoc P* = .000) and married (*post hoc P* = .029) patients, and significantly more frequent (*F*(3,296) = 4.02, *P* = .008) positive mental attitude than single patients (*post hoc P* = .009). Similarly, widowed patients revealed significantly more frequent (*F*(3,296) = 5.99, *P* = .001) health practices than single (*post hoc P* = .002) and divorced (*post hoc P* = .010) patients.

## Discussion

Health-promoting behaviors, such as appropriate nutritional habits, preventive actions or other healthy practices may be of importance in patients suffering from various chronic diseases [32], [33]. In our study, however, we did not find significant differences in health-promoting behavior between the subgroups of patients with different categories of diseases. Although a slight trend towards higher frequency of health-promoting behavior was observed for diabetes patients, this tendency disappeared after correction for multiple comparisons. This finding seems to point to the fact that the type of disease is not a major factor motivating patients to undertake health promoting behavior. This is probably against intuitive hypotheses which would allow predicting more behavioral health promoting effort in those categories of patients who have more control over the course of their disease (e.g. diabetes vs. neurologic patients). This can also be attributed to the fact that health-related behavior measured in our study was not disease-specific. Only few differences were observed between the clinical groups with respect to HLC, and these were limited only to patients with neurologic conditions who showed a significantly weaker belief in powerful others as a source of control over their health than did patients with diabetes and with the urinary system diseases. This finding may reflect lowered conviction in patients with neurologic disorders that powerful others (medical staff) can effectively manage their health [34], [35]. This may also be associated with a more generalized lowered sense of control over their disease, which may often be progressive and weakly responding to treatment [36]. No differences between the clinical samples in the levels of acceptance of illness suggest that the specificity of the disease-related burden may not be important in determining adjustment to the disease. In fact, other studies suggest that subjective factors such as personality or perceived social support may be more important for psychological adjustment than objective disease severity [37].

Interesting results were found in our study with respect to the associations between health-related behavior and HLC. In the total sample, the overall frequency of all types of health-related behavior (the total score on HRBI) was found to be positively related to both internal, powerful others and chance HLC, strikingly, with the highest correlation coefficients for powerful others HLC. This finding questions the universality of the claim that external HLC should be linked to unfavorable outcomes in health-related behavior [38], [39]. In this context, it is interesting to note that some studies also reported the associations between powerful others HLC and desirable health-related behavior. For instance, Steptoe et al. [18] found higher odds of attempts to quit smoking in those smokers who were higher on powerful others HLC. McConnell et al. [40] reported higher levels of powerful others HLC in the urban residents who managed to decrease cardiovascular risk after a psychoeducational intervention in comparison to those who did not, which suggests that powerful others HLC may enhance health-promoting behavior after such interventions. These authors also concluded that interventions aimed ate reduction of risk for a chronic disease should be more specific, taking into account sample-related factors. In another study, in a sample of patients with renal dialysis patients, greater perceived health competence was associated with more favorable adherence for the patients scoring low on internal and high on powerful others HLC [41]. We believe that the relatively strong positive association between powerful others HLC and all types of favorable health-related behaviors may be due to the sample effect – it should be noted that we examined patients with active chronic diseases whose motivation for health-related behavior may be more strongly affected by medical staff and depend on their confidence in health care providers than in non-clinical populations [41].

A more detailed analysis of the correlations between types of HLC and health-related behavior showed that the clinical subgroups differed with regard to the strengths and number of such associations. Powerful others HLC was a universal correlate of health-related behavior across all the subsamples, which provides more evidence power to this association. Inter-group differences were observed with respect to the associations between internal and chance HLC and health-related behavior. This suggests that disease-specific factors can be involved in the mediation of the relationship between HLC and health-related behavior. The contribution of such factors is still poorly understood, as most studies utilize clinically homogeneous samples, which makes inter-group comparisons difficult. However, certain clinical characteristics of chronic diseases, such as disease controllability, prognosis or disease-related burden were shown to be of relevance to adaptation, quality of life or other health outcomes [42], [43]. Our findings seem to indicate that positive associations between all types of HLC and favorable health-related behavior may be observed in a total sample of patients with various chronic diseases, however, in specific clinical samples some of these associations may be attenuated.

It is also of note that only one significant association was found in the total sample of our patients between health-related behavior and acceptance of illness, with higher acceptance related to lower frequency of preventive behavior. This suggests that higher acceptance of the disease-related burden may be a factor decreasing motivation for preventive actions. In this context, it may be relevant that some authors reported associations of higher acceptance of illness with more passive coping styles [44], which may also account for less initiative in undertaking preventive health-related behavior. This, however, may not hold true for certain conditions, as in one of our subsamples – patients with diabetes – acceptance of illness was found to correlate *positively* with a type of health-related behavior, namely healthy eating habits. This means that the patients with diabetes showing better acceptance of their disease burden are more inclined to healthy dietary behavior. One may speculate that this association is related to specific dietary regime required from patients with diabetes, and better adherence to this regime may results in less diabetes-related complications, thus making the disease more bearable, which translates in better acceptance of illness. The positive association between acceptance of illness and compliance with diet in patients with diabetes was actually found by Martin [45]. Of more importance here is probably a conclusion that the negative relationship between acceptance of illness and health-related behavior observed in the total sample of patients with chronic diseases can be reversed in some specific clinical populations, such as patients with diabetes.

We found that most sociodemographic variables were significantly associated with the frequency of at least some categories of health-related behavior. Gender effects were observed only with respect to nutritional habits, whereas age was positively related to all types of health-promoting behavior. More frequent healthy nutritional habits in women, as observed in our study, may reflect a more universal trend for women to be more aware and selective with regard to healthy foods. Increasing frequency of health promoting behavior with age can mirror increases in responsibility for health and in the value placed on health [46]. Similar results were reported by other authors. Women have consistently been found to reveal more healthy food choices than men [47], and positive correlations were also reported between age and several categories of health promoting behaviors [48]. Thus, our findings remain in accordance with the results of other studies, showing that age and gender are important determinants of health-related behavior [49].

Striking findings were observed in our study with respect to the effects of education on the frequency of health-related behavior. Generally, our patients with higher education showed less frequent health-promoting behavior than did patients with primary and vocational education. This may be in contrast with other reports that linked higher education to better health outcomes – the phenomenon known as the education gradient [50]. It should be noted, however, that we did not measure health status as an outcome variable, but rather health-related behavior, and the latter cannot be equaled with health status, especially in cross-sectional studies. Anyway, most studies point to the positive link between educational level and health-promoting behavior [51], therefore our findings should still be viewed with caution and need further corroboration. Marital status was found in our study to be significantly associated with health-related behavior, and, slightly surprisingly, widowed subjects showed more frequent health-promoting behavior. This effect, however, can most probably be attributed to the confounding effects of age. Since age and the widowed status are correlated, age-related increases in health-promoting behavior can overlap the frequency of this behavior reported by widowed subjects. In contrast to some other studies [52], we did not find differences in health-related behavior between those reporting different places of residence.

Overall, our study provided new empirical data on factors associated with frequency of health-related behavior among patients with various chronic diseases. The findings emphasize the complex network of possible factors affecting health-related behavior, and suggest that interventions aimed at health-related behavior modification should take into account these factors (e.g. gender, age, educational level, health-related beliefs).

It should also be noted that the choice of our sample might and probably did affect the findings we obtained. Most studies on health-related behavior have utilized non clinical population-based samples, and when clinical samples were investigated they were usually homogenous with respect to the clinical diagnosis. Our sample was markedly diverse with respect to the categories of diagnoses, as we wanted to search for generalized rather than disease-specific relationships. However, the inter-group differences we found for some of the associations warn us that the general tendencies between health-related behavior, HLC and acceptance of illness observed in the population of patients with chronic diseases as a whole may be attenuated, enhanced or even reversed in specific samples. Another important limitation of our study, which should be taken into account when analyzing the results, is a lack of precise control of depression and anxiety levels. Although patients with a history of major mental disorder were excluded from participation, this does not preclude that some patients may have had undiagnosed or subclinical levels of depression and anxiety, and this in turn might affect the results through introduction of an important source of variance into the scores of both health-related behavior and HLC. Future research should probably analyze the possible mediating effects of these mental conditions on the association between health-related behavior and HLC. Some of our findings (e.g. effects of education and HLC on health-related behavior) need definitely replication in other studies using clinically diverse samples similar to ours.

## Conclusions

Health-related behavior in patients with chronic diseases seems to be unrelated to a specific diagnosis. Both internal and external HLC, in particular powerful others HLC, were positively related to health-promoting behavior. Sociodemographic factors, including gender, age, education and marital status, are all factors determining frequency of health-related behavior in patients with chronic diseases.
